# Moving the Needle Toward Fair Compensation in Pediatric Nephrology

**DOI:** 10.3389/fped.2022.849826

**Published:** 2022-03-10

**Authors:** Darcy K. Weidemann, I. A. Ashoor, D. E. Soranno, R. Sheth, C. Carter, P. D. Brophy

**Affiliations:** ^1^Division of Nephrology, Children's Mercy Kansas City, Kansas City, MO, United States; ^2^University of Missouri-Kansas City School of Medicine, Kansas City, MO, United States; ^3^Division of Nephrology, LSU Health New Orleans and Children's Hospital, New Orleans, LA, United States; ^4^Departments of Pediatrics, University of Colorado, Bioengineering, and Medicine, Anschutz Medical Campus, Aurora, CO, United States; ^5^Department of Pediatrics, Loma Linda University Children's Hospital, Loma Linda, CA, United States; ^6^Division of Pediatric Nephrology, Rady Children's Hospital, University of California, San Diego, San Diego, CA, United States; ^7^Department of Pediatrics, University of Rochester School of Medicine, Rochester, NY, United States

**Keywords:** workforce, compensation, remuneration, pediatric nephrology, RVU

## Abstract

Remuneration issues are a substantial threat to the long-term stability of the pediatric nephrology workforce. It is uncertain whether the pediatric nephrology workforce will meet the growing needs of children with kidney disease without a substantial overhaul of the current reimbursement policies. In contrast to adult nephrology, the majority of pediatric nephrologists practice in an academic setting affiliated with a university and/or children's hospital. The pediatric nephrology service line is crucial to maintaining the financial health and wellness of a comprehensive children's hospital. However, in the current fee-for-service system, the clinical care for children with kidney disease is neither sufficiently valued, nor appropriately compensated. Current compensation models derived from the relative value unit (RVU) system contribute to the structural biases inherent in the current inequitable payment system. The perceived negative financial compensation is a significant driver of waning trainee interest in the field which is one of the least attractive specialties for students, with a significant proportion of training spots going unfilled each year and relatively stagnant growth rate as compared to the other pediatric subspecialties. This article reviews the current state of financial compensation issues plaguing the pediatric nephrology subspecialty. We further outline strategies for pediatric nephrologists, hospital administrators, and policy-makers to improve the landscape of financial reimbursement to pediatric subspecialists. A physician compensation model is proposed which aligns clinical activity with alternate metrics for current non-RVU producing activities that harmonizes hospital and personal mission statements.

## Introduction

The American Board of Pediatrics certified pediatric nephrology as a subspecialty in 1974. Since that time, 1,124 pediatric nephrologists have entered the field, with an average annual entry of 30–40 new graduates into the pipeline (1). Patient volumes in the field continue to rise, with significant growth over the last several decades due to improved survival of primary renal diseases as well as other non-renal conditions including congenital heart disease, childhood cancer, prematurity, and sepsis. Survival of these childhood illnesses is associated with increased incidence of chronic kidney disease (CKD) and/or hypertension in adolescence and young adulthood. The nation's obesity crisis is also driving an increase in the incidence of pediatric hypertension, the majority of whom are currently managed by pediatric nephrologists as routine hypertension management is not part of most pediatric residency training programs. In addition, many primary care physicians have less time devoted to managing complex or chronic conditions of any form, thereby driving patients with relatively mild disease into ongoing follow-up with pediatric nephrology. The cost of care for end stage kidney disease in the United States dwarfs the cost of any other single disease in the Medicare budget, accounting for $49.2 billion in expenses in 2018, the last year for which data are available (2). The majority of this goes to funding dialysis for adults, but the growing population of children with kidney disease will continue to propel this upward. A major opportunity to reduce this burden of illness and cost over the next several decades exists in early prevention of progressive kidney disease. This can only occur with skilled identification, treatment, and education of children at risk for kidney disease or with early kidney disease, all of which are best accomplished by the pediatric nephrologist. Despite this growing patient population, pediatric nephrology training programs are going unfilled as overall interest in the subspecialty wanes. We as a profession must all confront the reality that essential health care for children for kidney disease is not adequately valued, nor fairly compensated. Without significant changes to remuneration policies and financial incentives for underserved pediatric subspecialities such as pediatric nephrology, it is uncertain whether the profession will be able to meet the growing health care needs of children with kidney disease. This manuscript aims to describe the historical and multi-factorial causes of relatively imbalanced financial compensation issues in pediatric nephrology, as well as propose potential solutions designed to address the inequity.

## A Weakened Pipeline

Pediatric nephrology training remains one of the most stagnant professions of all the pediatric subspecialty workforce, with one of the highest unfilled training position rates amongst all specialties. Data from the American Board of Pediatrics Interactive Workforce database indicate that the pediatric nephrology trainee pipeline only increased on average by 0.5 or less trainees per year from 2001 to 2018 (3), which is in stark contrast to the fields of cardiology, neonatology, critical care medicine, and hematology-oncology who are experiencing rapid growth, and many more applicants than positions exist. The training program fill rates for pediatric nephrology have ranged from 41.5 to 73.9% over the past 5 years (4), with FY21 match data showing substantial improvement over prior years, although it is too early to determine if this is an anomaly due the COVID-19 pandemic or a true trendline. Preliminary analysis of the FY22 match revealed a more typical match rate of 55% (*n* = 33, 18 fewer matched fellows than the previous year), as well as 6 fewer positions available due to closure of fellowship programs. Similar trends have been observed amongst adult nephrology programs (5, 6). Moreover, there are higher attrition rates observed in pediatric nephrology training programs, with upwards of 22% of trainees not completing their training (7). A staggering 33% of pediatric nephrologists planned to reduce or stop pediatric nephrology clinical activities over the next 5 years (8) due to retirement or dissatisfaction with work-life balance or compensation. Additionally, although it is difficult to determine the true quantity of practicing clinical FTE of a workforce, reports indicate that a substantial minority of board-certified pediatric nephrologists are not practicing nephrology, with one large survey of 183 pediatric nephrology graduates over the previous 10 years indicating that 35 (19%) were not practicing pediatric nephrology in the United States (9). These disheartening trends lead one to question whether there are enough board-certified pediatric nephrology training graduates just to replace those leaving the profession, notwithstanding speculation about further growth of the workforce.

An understanding of why trainees do not choose pediatric nephrology is necessary to develop strategies for addressing the shrinking workforce. A 2008 survey of pediatric nephrology fellows found that lack of interest in the subject matter was the most commonly cited reason why their colleagues did not choose pediatric nephrology as a profession, although the second most cited reason was due to financial burden, and the third was due to perceived high workload (10). Similar data was reported in a 2010 survey of 103 pediatric nephrology fellows who reported intense workloads and financial burden as important deterrents toward interest in the field, with more than 40% of fellows reporting 75 h or more per week at work while on clinical service rotations (7). Another survey (9) of non-renal pediatric fellows interestingly found that “monetary benefit is not adequate” was only reported in 13% of trainees, and was the least common concern to cite and rated below other factors such as lack of role models (25%), too difficult subject matter (22%), or poor lifestyle (18%). However, another survey of medical students who chose not to go into nephrology found that 43% of individuals were concerned by negative remuneration (11). Negative perceptions of the workload compared to the rewards (financial or otherwise) remain persistent among trainees and pediatric nephrologists.

The salary discrepancy between pediatric nephrologists and other physicians is likely a major deterrent to choosing a career in pediatric nephrology, especially for students who carry large educational debt. Catenaccio et al. (12) found that pediatric nephrologists incurred a > $750,000 deficit in lifetime earnings by choosing a career in pediatric nephrology over general pediatrics. The same research group also found that on average, adult physicians make ~ 25% more than pediatricians; for nephrology the difference in lifetime earnings between adult and pediatric nephrologists is around $1.2 million dollars over a 30-year career (13). In 2020 the American Association of Medical Colleges (AAMC) reported that 73% of medical school graduates report educational debt, with a median value of $300,000 (14). Financially-savvy trainees interested in pediatric nephrology, therefore, may view the financials of a relatively prolonged 10-year training period coupled with low pay and potential geographic restrictions in job availability with substantial trepidation, even if they may have initially been attracted to the subspecialty through their clinical exposure. Indeed, this problem is not limited to pediatric nephrology, but similar workforce issues plague other poorly-compensated cognitive specialties such as rheumatology, endocrinology, infectious diseases, and adolescent medicine. Although certainly the reasons for choosing a specialty are not limited to the financial implications, it is likely no coincidence that a steeply linear relationship exists between fellowship match rates and earning potential (15).

## A Fundamentally Flawed Reimbursement System

Inequities in physician salaries are multifactorial, although much of the inequity particularly for cognitive-based specialties like pediatric nephrology stems from long-standing structural biases inherent in the traditional payment model in the United States. The most widely used measures of clinical productivity in the United States stem from the Relative Value Unit (RVU) system which assigns physician “work” relative to the Current Procedural Terminology (CPT) diagnostic and billing codes (16). CPT codes are used to describe medical, surgical, and diagnostic services in uniform language amongst physicians, medical billing, and payers for financial and administrative purposes. RVUs consist of three components: *physician work RVUs* (wRVU) which account for the time, technical effort and skill, medical decision-making, and mental stress required to provide a certain service; *practice expense RVUs* which accounts for the nonphysician labor, equipment, and building space; and *professional liability insurance RVUs* which factor in the costs of malpractice premiums. Although RVUs themselves are not monetary values, they can be multiplied by a conversion factor (dollars per RVU) to determine the amount of payment for a service. Since 1992, the Centers for Medicare and Medicaid (CMS) and most private payers utilize the RVU system to pay physicians in the United States. At the time it was developed, the RVU system was felt to improve upon the prevailing “usual, customary, and reasonable” models of reimbursement based on local community standards. Over time, RVU values were updated and refined, and are mandated by Congress to be updated no less than every 5 years. Importantly, a component of “net neutrality” is incorporated into the revision to prevent escalating health care costs, with any increase in a RVU service amount automatically resulting in a decrease in RVU valuation for another clinical service. In 2021 CMS made the changes to the CPT coding criteria for outpatient visits to include time spent on the day of the clinic visit in an attempt to capture some of the previous uncompensated time spent on cognitive work for the non-procedural specialist. The results of this intervention on physician billing have not yet been compiled or reported, but may provide a corrective step for pediatric nephrologists.

The wRVU system is particularly problematic for pediatric nephrology. Pediatric nephrology consists of both cognitive and procedural components. A special emphasis on primary care components exists, which often requires multi-disciplinary collaboration with other specialists and primary care physicians to address multiple complex comorbidities and preventative care aimed at slowing progression of kidney disease. Unlike other preventative and primary care specialists, however, pediatric nephrologists are required to provide 24-h emergency call, often with the provision of emergent dialysis for critically-ill patients and kidney transplantation. Acute dialysis requires physician presence during treatment for billing purposes which is unique to the field. Furthermore, the ability to provide acute renal replacement modalities is imperative for the support of key money-making service lines of a children's hospital (i.e., cardiac surgery and intensive care, transplant services, and level 4 NICU). However, the pay scale for pediatric nephrology salaries is more closely aligned with other cognitive pediatric subspecialties like rheumatology, infectious diseases, and endocrinology which often have much less onerous after-hours call burdens. On the other hand, our critical care, hospitalist, emergency medicine, and neonatology colleagues who have similar night call expectations do not have outpatient clinical responsibilities when their coverage shifts are complete. The compensation for the perceived workload and “uncompensated” call therefore adversely affects trainee interest in pursuing a nephrology career, as well as leading to attrition within the specialty as more senior pediatric nephrologists seek non-clinical career advancements that alleviate the substantial burden of night and weekend call obligations.

As most physicians are painfully aware, wRVU have become the dominant currency of productivity and compensation metrics which are now benchmarked within subspecialties through member-driven healthcare administrative companies such as the MGMA, AAAP, AAMC, and SullivanCotter. Many believe that wRVUs are widely misvalued, and unfairly favor procedure-based specialties (17). The relative allocations have not kept pace with the technological advances achieved over the last several decades allowing for more efficient procedures to yield high substantially higher wRVUs for a given amount of time. In contrast, the so-called technological “advancements” for cognitive specialties have trapped physicians in a low-level office-based clinic wRVU setting encumbered by increased chart review and documentation requirements within the electronic medical record (EMR). This may be particularly so in pediatric nephrology which requires management (and documentation of such) for up to 8–10 co-morbidities. A large-scale time motion study (18) found that adult physicians spent an average of 16 min and 14 s per encounter interacting with the EMR. A similar pediatric study (19) found that pediatric nephrologists spend an average of 17.7 min per encounter; the 4^th^ longest time, after the other poorly-compensated cognitive specialties (endocrinology 19.7 min, infectious diseases 20.8 min, and rheumatology 26.4 min).

Pediatric nephrology is unique amongst all pediatric specialties that their patients may receive Medicare benefits, as well as Medicaid. The Medicare Kidney Disease Entitlement Social Security Amendments of 1972 (20) provides Medicare benefits to all adults and children under the age of 18 with ESKD, and represents the only pediatric demographic that may receive such entitlements. Ongoing advocacy efforts by various societies including the Renal Physicians Association (RPA) have yielded laudable improvements in the 2021 Medicare Fee Schedule proposed rule which increased reimbursement for outpatient dialysis codes, although notably there was a smaller overall percentage increase in the pediatric codes as compared to the adult codes by more 50% (Table 1) (21). Management of children on dialysis requires significantly more time and attention that managing adult dialysis patients due to the overlying complexities of development, growth, nutrition, and school performance as well as the underlying disease processes. Although Medicaid is jointly funded by federal and state funding, the flexibility allowed by individual state administration has led to significant variability amongst the nation's various Medicaid programs (22). The federal government pays for 60% of total Medicaid costs, with the remainder paid by for the states. Even within a state, managed care organizations may have different approaches and different state law allocations. One recent study found that public payers such as Medicaid more frequently underpaid children's hospitals which are an important safety net for underprivileged children, with 51% of admissions underpaid by Medicaid compared to 18% for private payers (23). Children of color and those with disabilities are disproportionately represented by Medicaid as opposed to private health insurers. We know that these underrepresented groups are more susceptible to kidney disease over the course of their lifetime, making this a critical issue specific to nephrology. Underpayment by Medicaid makes it harder for these groups to achieve equity in health care access and delivery, thereby contributing to ongoing structural racism in healthcare delivery.

**Table 1:** Comparison of selected adult and pediatric ESKD CPT codes.

**CPT code**	**CY2020 wRVUs**	**CY2021 wRVUs**	**% increase**	**Corresponding adult CPT code**	**CY2020 wRVUs**	**CY2021 wRVUs**	**% increase**
90951 (in-center HD <2 y, 4+ visits)	18.46	23.92	29.6%	90960 (in-center HD, >20 y, 4+ visits)	5.18	6.77	30.6%
90954 (in-center HD, 2–11 years, 4+ visits	15.98	21.44	34.2%				
90957 (in-center HD,12–19 y. 4+ visits)	12.52	15.46	23.3%				
90955 (in-center HD, 2–11 y, 2–3 visits)	8.79	13.32	17.4%	90961 (in-center HD, >20 y, 2–3 visit)	4.26	5.52	29.6%
90958 (in-center HD, 12–19 y, 2–3 visits)	8.34	9.87	18.3%				
90956 (in-center HD, 2–11 y, 1 visit	5.95	6.64	11.6%	90962 (in-center HD, > 20 y, 1 visit)	3.15	3.57	13.3%
90959 (in-center HD, 12–19 y, 1 visit	5.5	6.19	12.5%				
90963 ESRD home <2	10.56	12.09	14.5%	90966 (ESRD home dialysis > 20)	4.26	8.04	88.7%
90964 ESRD home 2–11	9.14	10.25	12.1%				
90965 ESRD home 12-19	8.69	9.8	12.8%				
Average % increase			18.6%				40.6%

*Source: https://www.cms.gov/files/document/cms-1734-p-pdf.pdf. Accessed October 14, 2021*.

In general, pediatricians in private practice earn more than pediatricians employed by health systems. Although the reasons may be multi-faceted, simply-put, private practices do not rely on benchmarking wRVU for compensation. In practice models based on full partnership, physician compensation is instead determined by an “eat-what-you-kill” philosophy: overall collections minus overhead. However, these solo or small-group practice models are a rarity in the field of pediatric nephrology. The majority (73%) of pediatric nephrologists are employed in an academic setting, with the remainder working in a pediatric, specialty, or multi-specialty practice (17%), community hospital (4%), or solo private practice (1%) (8). RVUs and salaries are benchmarked nationally and used subsequently for physician contracts for most academic settings and private practices integrated into large health care systems. Such salary offers are often obscure and favor the negotiation powers of the institution over the individual, thereby leading an individual to accept a salary offer below fair market value due to ignorance or perceived lack of negotiation power. This perpetuates the chronic devaluation of the evolving work of pediatric nephrologists and further weakens the bargaining power of pediatric nephrologists who attempt to mitigate these inequities at a local or institutional level.

## A Field of Unequitable Compensation and Opportunities

One cannot discuss physician compensation issues without discussion of the racial, ethnic, and gender gaps in compensation. Women make up over half of medical school graduates, and represented 80% of the most recently certified pediatric nephrologists in 2020 (1). A recent AAMC report (24) from a national analysis of AAMC's annual Faculty Salary Survey which encompasses academic payscales of > 60,000 physicians found that, with rare exceptions, females and black, indigenous, and people of color (BIPOC) faculty were paid significantly less than their male and/or white counterparts, even after accounting for degree, specialty, and rank. For academic pediatric subspecialists, white women make $0.85 for every $1.00 made by a white man; for BIPOC faculty the pay gap ranged from $0.70 for American Indian or Alaskan native; $0.84 for Asian, $0.84 for black or African American, $0.80 for Latino, and $0.89 for Native Hawaiian or Other Pacific Islander. Unfortunately the data have never been published specifically for pediatric nephrology, but given the uniformity of findings across all other specialties we doubt it would be more equitable within the field of pediatric nephrology. Disappointingly, despite greater scrutiny regarding disparities in physician pay over recent years, the report also found few substantial changes to the disparities observed over a 5-year period from FY2013 to FY2017. Similar trends have been observed for pediatricians (25) and adult nephrology (26); in fact, adult nephrology was found to have the largest pay discrepancy of > $16K for any of the adult specialties even after adjustment for experience, productivity, and hours worked.

The reasons for why such pay discrepancies exist are multi-factorial and engrained in a traditional compensation system in desperate need of an overhaul. The traditional compensation model for employees includes a formula for base salary (using national benchmark data) with additional rewards for productivity, seniority, and leadership. The structural inequities at every level of compensation perpetuate further disparity, as earning potential is diminished in all potential areas of compensation (27). A significant difference often exists between the low and high end of base-salary wages during the initial hiring phase, with less successful negotiations well documented amongst women physicians (28). Productivity-based incentives may be negatively affected by increased organizational and service demands, roles more often filled by women. Women in nephrology historically have had fewer opportunities for formal leadership roles or promotion opportunities (29) which translates into less compensation.

Somewhat unique to the pediatric nephrology specialty is the relative proportion of international medical graduates (IMG), which constitute around 40% of the U.S. pediatric nephrology workforce. Although IMG are often offered opportunities for training and professional development beyond what is often available in their countries of origin, IMG physician encounter substantial barriers in the transition from training to practice and early-stage career development (4). These physicians must navigate incredibly complex immigration policies and stringent visa requirements to maintain their legal standing and ability to practice medicine in the United States. Individuals on a J-1 visa must abide by the “home rule” requirement which requires individuals to leave the US for a 2-year period after training. The only way to circumvent this requirement is to apply for a “J-1 Waiver” position in an underserved area, which are typically primary care positions and are rarely offered by academic institutions and/or children's hospitals which are the majority of employers for pediatric nephrology. The requirements for more senior physicians with advanced training overseas are even more draconian, as fellows who have not completed residency in the US cannot sit for the subspecialty boards until they have completed the General Pediatrics boards, with a 10-year maximum duration training window to achieve certification. This requirement highly disincentivizes pediatric nephrology training programs from even considering applicants who have not completed a US residency. Qualified IMG applicants interested in pursuing a career in pediatric nephrology then typically must repeat a pediatrics residency in the US before they can apply for fellowship positions. Recent match statistics (30) have revealed another potentially concerning trend with plummeting interest in applications from IMG physicians who traditionally have filled the vacant positions due to ongoing lack of interest from US medical graduates (USMG). IMGs who do pursue training in pediatric nephrology often face significant restrictions on the geographic locale or types of jobs available with their visa requirements. In the American Society of Nephrology 2019 Nephrology Fellow Survey, a majority 64% of pediatric IMG fellows reported dissatisfaction with the local job market (31). Overall, IMGs reported more difficulty attaining “satisfactory” post-fellowship positions compared with US USMG (48.9 vs. 27.1%, respectively). For IMG interested in a significant research career, they are typically dependent on a much smaller grant pool (mostly from professional societies or institutional grants) of which to apply for researching funding, as most NIH grants are limited to US citizens and permanent residents.

## Potential Solutions to Promote Meaningful Change

### Collective Bargaining Power

A number of potential strategies exist to remediate the substantial barriers regarding fair compensation. The collective bargaining power of pediatric subspecialist-specific professional societies should be harnessed, to ensure that efforts to promote meaningful change within the public policy arena are amplified. We can model our strategy after the efforts of other undervalued medical professions who have achieved specialty-specific increases in physician compensation through legislative advocacy, such as the American Geriatric Society (32) who successfully advocated for innovative payment codes that benefitted geriatric subspecialties. We must align with the American Society of Nephrology who recently committed a task force (33) designed to improve nephrologist compensation and resolved to launch concrete, transparent efforts to reduce bias and improve the data systems that feed into physician benchmarking compensation and productivity surveys. It is crucial that the voice of the small but mighty pediatric nephrology community is not diminished during this ongoing work.

### Advocacy

In 2009 The John E. Lewy Foundation (JELF) and the American Society of Pediatric Nephrology collaborated to develop an Advocacy Scholars Program to provide mentored advocacy training to pediatric nephrology fellows and to establish ongoing relationships with government officials for policy work (34). This initiative demonstrates the importance of legislative advocacy through harnessing the collective voices and bargaining power of pediatric subspecialty societies. The addition of formalized advocacy training within the training environment may leverage pediatric nephrologists to better support change in these areas in the future. As pediatric subspecialists, we must continue staunch advocacy efforts to achieve compensation parity between Medicaid and Medicare. Although the Affordable Care act temporarily funded payment parity for 2 years in 2013–2014, ongoing legislation and advocacy efforts to ensure permanent payment parity should be reinstated at the federal level. Current bills under consideration in Congress includes The Kids Access to Primary Care Act, H.R. 6159 and The Ensuring Access for Women's and Children's Act, S. 4088 which all represent potential steps in the right direction.

Pediatric subspecialists must demand greater representation when compensation schemes are determined locally and nationally. CMS determines the physician fee schedule for RVUs based in part on the input of the multi-specialty Relative Value Scale Update Committee that is composed of members from 26 different specialties (35), but only includes one pediatric representative on this committee. Although pediatric subspecialists may in theory be represented by their adult counterparts on the committee, the care of children with kidney disease varies greatly compared to adults with kidney disease; the current system of representation may therefore overvalue services predominantly performed by adults.

Leveraging improvements in telehealth and use of digital technology may also improve the remuneration landscape for pediatric nephrologists. Many health care systems have incorporated telephone or electronic consultation systems in which subspecialists provide peer-to-peer consultations, which may encourage primary care physicians to manage problems that would otherwise have been referred to subspecialty care (36), although currently time spent by the subspecialist on these services is not compensated. CMS recently approved billing codes to support services such as these, although they have not yet been adopted by public and private payers.

### Enhanced Loan Mitigation Programs

Loan mitigation programs designed to defray the substantial economic burden incurred by trainees entering the pediatric nephrology workforce are paramount to ensuring a healthy pipeline. A host of subspecialty organizations have routinely called on Congress to fund the Pediatric Subspecialty Loan Repayment Program (PSLRP) which would provide up to $35,000 annually for up to 3 years to pediatric subspecialists who agree to practice in an underserved area. The PSLRP was reauthorized into law by Congress for 5 years with the passage of the Coronavirus Aid, Relief, an Economic Security (CARES) Act in March 2020 although was not included in the final FY21 federal omnibus spending bill in December 2020. Recently ASN has committed $2.7 million toward a pilot 3-year loan mitigation program for trainees entering nephrology with a special focus on those from underrepresented racial minorities. Many pediatric nephrology faculty positions may be eligible for forgiveness through the Public Service Loan Forgiveness program which was established by Congress in 2007 to allow certain professionals working in the public service and nonprofit sectors under qualifying income-based repayment programs to receive forgiveness of federal Direct Loans after 10 years of qualifying work. Growing recognition of the program despite a thus far rather lackluster implementation has led to an increase in trainees making career decisions based on PLSF eligibility (37), although only ~14% of students were able to correctly identify all of the necessary criteria to successfully qualify for forgiveness. Formal training and educational lectures, given by subspecialty societies and/or medical schools, designed to improve awareness of potential loan forgiveness programs may enhance pipeline efforts with potential trainees. Broadening the criteria for loan forgiveness to include subspecialties like pediatric nephrology, where a workforce shortage is predicted in the next decade, could also help to attract trainees to the specialty. Ongoing targeted advocacy for support of these crucial loan forgiveness programs is a key item on the JELF Scholar's legislative agenda for the coming year. If more pediatric nephrologists can be engaged to write letters to their own elected representatives, our amplified voice may be able to finally achieve successful negotiations for a federal pediatric subspecialist loan forgiveness program during upcoming budget negotiations.

### Progressive Compensation Models

**Figure 1:**
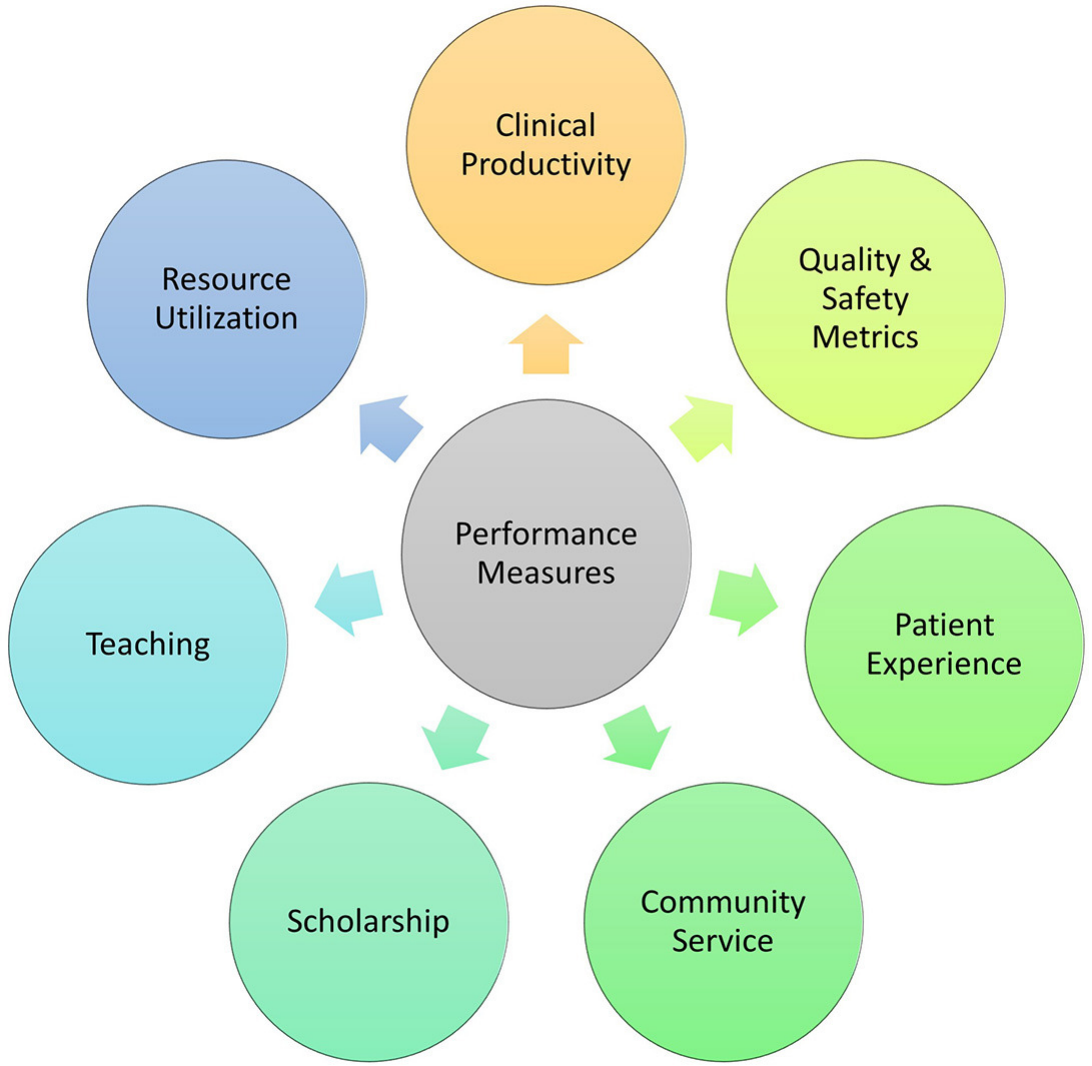
Proposed compensation model for pediatric nephrology. Compensation models for pediatric nephrology can incorporate other performance measures beyond clinical productivity data to measure and reward academic productivity. Additional performance measures that can be developed and assessed with pre-defined metrics of success include quality and safety metrics, patient experience, community service, scholarship, teaching, and resource utilization.

Innovative compensation models that incentivize quality and value care, over volume (wRVU) are fundamental to improving the financial remuneration of pediatric nephrologists. Productivity goals that rely solely on wRVU are fatally flawed, and do not serve the individual values of clinicians nor the greater mission of society to improve the health and wellbeing of children. Transcendent compensation models that more accurately capture the work of pediatric subspecialists recognize clinical work, in addition to meeting other important mission of academic institutions of research, education, and service (Figure 1). As one example, Mezrich and Nagy described the concept of an “academic RVU” which can be used as an adjunctive metric to track non-clinical work including publications, educational scholarship and teaching, an administrative and service roles (38). Giacoma et al. (39) describe a customized RVU (cRVU) system for transplant nephrologists and surgeons which is a value-based compensation model that also incorporates non-billable work and captures the entire spectrum of clinical, academic, and relationship-building efforts necessary for a robust, high-value transplant program. Similar customization could be designed for other complex aspects of pediatric nephrology care that directly affect the long-term risks of more severe kidney disease including acute kidney injury prevention, vasculitis management, and hypertension programs. Furthermore, such models could also incorporate a bonus system for taking evening and weekend call, like emergency medicine or intensive care, which both incentivizes and appropriately compensates the relatively intense call burden that is necessary to provide adequate 24-h coverage to support the clinical needs of contemporary children's hospitals. Progressive compensation models may also allow for development of pediatric nephrology programs in small group settings to sustain longevity of a solo and/or small-group practice in a more rural community-hospital setting which would address the geographic disparities faced by many patients who must travel several hundred miles or more to receive care.

### Promote a Supportive Environment

An inclusive, supportive work environment is necessary to ensure the sustainability of a 30-plus year career in pediatric nephrology. As the millennial generation of physician trainees enters into the labor force, there is a greater emphasis on flexible work environments. Greater leadership support for nontraditional careers such as part-time work, job-shares, and relaxed promotion timelines will likely promote retention and spark interest from trainees who may place a greater value on shift work to allow adequate time for pursuits outside of medicine. Although burnout is pervasive within all areas of medicine, a 2020 pilot study examining burnout in pediatric nephrology trainees and faculty found a much lower than expected prevalence of burnout of only 16% amongst faculty and 13% in trainees (40). These notes of optimism should be celebrated amongst the pediatric nephrology community and shared with potential recruits to generate interest in the field. One of the greatest strengths of the pediatric nephrology community arises from the collaborative efforts that include physicians and trainees at all levels. All pediatric nephrologists have a duty to our current and future patients and colleagues to provide supportive training environments for trainees, and role model a career that embraces meaningful and sustained work-life integration.

## Conclusions

We are at a crossroads moment that will determine the sustainability of our pediatric nephrology workforce. As we have highlighted, significant and widespread financial and remuneration challenges currently exist that threaten our pipeline and existing workforce, and portend a future at risk of inadequate coverage for exponentially growing complexity and demand. There is no quick fix to any of the issues discussed herein. Solutions will likely require concerted efforts to bring about a culture change in health care delivery and reimbursement models to pave the way toward improved valuation of the unique services provided by a pediatric nephrologist. Pediatric nephrology, through its professional organizations and partners will need to mobilize resources to identify common goals and highlight potential opportunities to leverage policy changes designed to better reflect the value of care we provide to children with kidney disease. Doing so will require broad participation, multi-disciplinary consensus-building, creativity, and likely a fair amount of tenacity. Although significant systemic and policy changes are certainly necessary, we also have a collective responsibility on an individual grassroots level to nurture the next generation of pediatric nephrologists. This rewarding career path affords intellectual stimulation, a diverse array of patients, and a unique mix of acute inpatient care combined with long-term longitudinal care which allows for meaningful connections with patients and their families. An improved valuation of the highly impactful role a pediatric nephrologist serves in improving the lives of children with kidney disease will serve to preserve the workforce for generations to come.

## Author Contributions

DW conceptualized the manuscript, wrote the first draft, reviewed revisions, and finalized the manuscript. IA, DS, RS, CC, and PB reviewed and revised the manuscript. All authors contributed to the article and approved the submitted version.

## Conflict of Interest

The authors declare that the research was conducted in the absence of any commercial or financial relationships that could be construed as a potential conflict of interest.

## Publisher's Note

All claims expressed in this article are solely those of the authors and do not necessarily represent those of their affiliated organizations, or those of the publisher, the editors and the reviewers. Any product that may be evaluated in this article, or claim that may be made by its manufacturer, is not guaranteed or endorsed by the publisher.
